# Supplementary material for: Measuring walking impairment in patients with intermittent claudication: psychometric properties of the Walking Estimated-Limitation Calculated by History (WELCH) questionnaire
Source: PeerJ. 2021 Aug 26;9:e12039. doi: 10.7717/peerj.12039 (PMC8415277; doi:10.7717/peerj.12039)
Supplement: Supplemental Information 1 [file peerj-09-12039-s001.docx]

|  | **Baseline** | | | |  | | **Lost to 12-months follow-up** | | |  | | **Followed through 12 months** | | | |  | **12-month follow-up*** | | |
| --- | --- | --- | --- | --- | --- | --- | --- | --- | --- | --- | --- | --- | --- | --- | --- | --- | --- | --- | --- |
| **Sociodemographic characteristics** | **TeGeCoach (n=984)** | **Routine Care (n=987)** | **Total (n=1971)** |  | | **TeGeCoach** | | **Routine Care** | **Total** | |  | | **TeGeCoach** | **Routine Care** | **Total** |  | **TeGeCoach** | **Routine Care** | **Total** |
| ***N. of questionnaires received*** | 811 | 885 | 1696 |  | | 281 | | 270 | 551 | |  | | 530 | 615 | 1145 |  | 588 | 645 | 1233 |
| ***Sex ^a^*** |  |  |  |  | |  | |  |  | |  | |  |  |  |  |  |  |  |
| *Female* | 248 (30.6) | 281 (31.8) | 529 (31.2) |  | | 100 (35.6) | | 94 (34.8) | 194 (35.2) | |  | | 148 (27.9) | 187 (30.4) | 335 (29.3) |  | 160 (27.2) | 199 (30.9) | 359 (29.1) |
| *Male* | 549 (67.7) | 597 (67.5) | 1146 (67.6) |  | | 178 (63.3) | | 174 (64.4) | 352 (63.9) | |  | | 371 (70.0) | 423 (68.8) | 794 (69.3) |  | 417 (70.9) | 441 (68.4) | 858 (69.6) |
| *No information provided* | 14 (1.7) | 7 (0.8) | 21 (1.2) |  | | 3 (1.1) | | 2 (0.7) | 5 (0.9) | |  | | 11 (2.1) | 5 (0.8) | 16 (1.4) |  | 11 (1.9) | 5 (0.8) | 16 (1.3) |
| ***Age (in years) ^b^*** | 66.4 (8.6) | 66.3 (8.6) | 66.3 (8.6) |  | | 65.2 (9.4) | | 63.9 (8.9) | 64.6 (9.2) | |  | | 67.0 (8.2) | 67.4 (8.3) | 67.2 (8.2) |  | 67.1 (8.3) | 67.3 (8.4) | 67.2 (8.3) |
| *Minimum - Maximum* | 35-81 | 38-81 | 35-81 |  | | 38-81 | | 39-81 | 38-81 | |  | | 35-81 | 38-81 | 35-81 |  | 35-81 | 38-81 | 35-81 |
| ***BMI ^b^*** | 28.1 (5.3) | 28.1 (4.8) | 28.1 (5.0) |  | | 28.4 (5.2) | | 28.3 (4.9) | 28.4 (5.1) | |  | | 27.9 (5.4) | 28.0 (4.7) | 27.9 (5.0) |  | 27.8 (5.3) | 28.0 (4.7) | 27.9 (5.0) |
| *Minimum-Maximum* | 15.0-75.8 | 17.0-45.2 | 15.0-75.8 |  | | 19.4-54.9 | | 18.3-45.2 | 18.3-54.9 | |  | | 15.0-75.8 | 17.0-44.5 | 15.0-75.8 |  | 14.5-75.8 | 16.8-44.5 | 14.5-75.8 |
| ***Education ^b^*** *(multiple choices possible)* |  |  |  |  | |  | |  |  | |  | |  |  |  |  |  |  |  |
| *Apprenticeship* | 553 (68.2) | 613 (69.3) | 1166 (68.8) |  | | 188 (66.9) | | 182 (67.4) | 370 (67.2) | |  | | 365 (68.9) | 431 (70.1) | 796 (69.5) |  | 365 (62.1) | 431 (66.8) | 796 (64.6) |
| *College* | 265 (32.7) | 297 (33.6) | 562 (33.1) |  | | 88 (31.3) | | 73 (27.0) | 161 (29.2) | |  | | 177 (33.4) | 224 (36.4) | 401 (35.0) |  | 177 (30.1) | 224 (34.7) | 401 (32.5) |
| *University* | 140 (17.3) | 149 (16.8) | 289 (17.0) |  | | 35 (12.5) | | 45 (16.7) | 80 (14.5) | |  | | 105 (19.8) | 104 (16.9) | 209 (18.3) |  | 105 (17.9) | 104 (16.1) | 209 (17.0) |
| *Other* | 68 (8.4) | 58 (6.6) | 126 (7.4) |  | | 30 (10.7) | | 29 (10.7) | 59 (10.7) | |  | | 38 (7.2) | 29 (4.7) | 67 (5.9) |  | 38 (6.5) | 29 (4.5) | 67 (5.4) |
| *No education* | 44 (5.4) | 23 (2.6) | 67 (4.0) |  | | 22 (7.8) | | 9 (3.3) | 31 (5.6) | |  | | 22 (4.2) | 14 (2.3) | 36 (3.1) |  | 22 (3.7) | 14 (2.2) | 36 (2.9) |
| ***Income ^b^*** |  |  |  |  | |  | |  |  | |  | |  |  |  |  |  |  |  |
| *< 500€* | 16 (2.0) | 18 (2.0) | 34 (2.0) |  | | 11 (3.9) | | 6 (2.2) | 17 (3.1) | |  | | 5 (0.9) | 12 (2.0) | 17 (1.5) |  | 5 (0.9) | 12 (1.9) | 17 (1.4) |
| *500€ to 1000€* | 63 (7.8) | 72 (8.1) | 135 (8.0) |  | | 30 (10.7) | | 31 (11.5) | 61 (11.1) | |  | | 33 (6.2) | 41 (6.7) | 74 (6.5) |  | 33 (5.6) | 41 (6.4) | 74 (6.0) |
| *1001€ to 1500€* | 101 (12.5) | 114 (12.9) | 215 (12.7) |  | | 43 (15.3) | | 45 (16.7) | 88 (16.0) | |  | | 58 (10.9) | 69 (11.2) | 127 (11.1) |  | 58 (9.9) | 69 (10.7) | 127 (10.3) |
| *1501€ to 2000€* | 137 (16.9) | 145 (16.4) | 282 (16.6) |  | | 52 (18.5) | | 31 (11.5) | 83 (15.1) | |  | | 85 (16.0) | 114 (18.5) | 199 (17.4) |  | 85 (14.5) | 114 (17.7) | 199 (16.1) |
| *2001€ to 2500€* | 153 (18.9) | 153 (17.3) | 306 (18.0) |  | | 45 (16.0) | | 50 (18.5) | 95 (17.2) | |  | | 108 (20.4) | 103 (16.7) | 211 (18.4) |  | 108 (18.4) | 103 (16.0) | 211 (17.1) |
| *2501€ to 3000€* | 110 (13.6) | 132 (14.9) | 242 (14.3) |  | | 31 (11.0) | | 37 (13.7) | 68 (12.3) | |  | | 79 (14.9) | 95 (15.4) | 174 (15.2) |  | 79 (13.4) | 95 (14.7) | 174 (14.1) |
| *3001€ to 3500€* | 64 (7.9) | 83 (9.4) | 147 (8.7) |  | | 22 (7.8) | | 16 (5.9) | 38 (6.9) | |  | | 42 (7.9) | 67 (10.9) | 109 (9.5) |  | 42 (7.1) | 67 (10.4) | 109 (8.8) |
| *3501€ and more* | 99 (12.2) | 112 (12.7) | 211 (12.4) |  | | 26 (9.3) | | 34 (12.6) | 60 (10.9) | |  | | 73 (13.8) | 78 (12.7) | 151 (13.2) |  | 73 (12.4) | 78 (12.1) | 151 (12.2) |
| *No information provided* | 68 (8.4) | 56 (6.3) | 124 (7.3) |  | | 21 (7.5) | | 20 (7.4) | 41 (7.4) | |  | | 47 (8.9) | 36 (5.9) | 83 (7.3) |  | 105 (17.9) | 66 (10.2) | 171 (13.9) |
| ***Marital status ^b^*** |  |  |  |  | |  | |  |  | |  | |  |  |  |  |  |  |  |
| *Single* | 53 (6.5) | 60 (6.8) | 113 (6.7) |  | | 24 (8.5) | | 24 (8.9) | 48 (8.7) | |  | | 29 (5.5) | 36 (5.9) | 65 (5.7) |  | 29 (4.9) | 36 (5.6) | 65 (5.3) |
| *Married* | 525 (64.7) | 563 (63.6) | 1088 (64.2) |  | | 159 (56.6) | | 151 (55.9) | 310 (56.3) | |  | | 366 (69.1) | 412 (67.0) | 778 (67.9) |  | 366 (62.4) | 412 (63.9) | 778 (63.1) |
| *Divorced/separated* | 140 (17.3) | 157 (17.7) | 297 (17.5) |  | | 63 (22.4) | | 58 (21.5) | 121 (22.0) | |  | | 77 (14.5) | 99 (16.1) | 176 (15.4) |  | 77 (13.1) | 99 (15.3) | 176 (14.3) |
| *Widowed* | 77 (9.5) | 90 (10.2) | 167 (9.8) |  | | 31 (11.0) | | 29 (10.7) | 60 (10.9) | |  | | 46 (8.7) | 61 (9.9) | 107 (9.3) |  | 46 (7.8) | 61 (9.5) | 107 (8.7) |
| *No information provided* | 16 (2.0) | 15 (1.7) | 31 (1.8) |  | | 4 (1.4) | | 8 (3.0) | 12 (2.2) | |  | | 12 (2.3) | 7 (1.1) | 19 (1.7) |  | 70 (11.9) | 37 (5.7) | 107 (8.7) |
| ***Number of children ^a^ **** | 1.8 (1.1) | 1.6 (1.1) | 1.7 (1.1) |  | | 1.8 (1.2) | | 1.5 (1.1) | 1.7 (1.1) | |  | | 1.8 (1.1) | 1.7 (1.2) | 1.7 (1.1) |  | 1.8 (1.1) | 1.7 (1.2) | 1.7 (1.1) |
| *Minimum-Maximum* | 0-7 | 0-11 | 0-11 |  | | 0-7 | | 0-7 | 0-7 | |  | | 0-6 | 0-11 | 0-11 |  | 0-6 | 0-11 | 0-11 |
| ***Profession ^b^*** *(multiple choices possible)* |  |  |  |  | |  | |  |  | |  | |  |  |  |  |  |  |  |
| *Employed* | 227 (28.0) | 235 (26.6) | 462 (27.2) |  | | 84 (29.9) | | 94 (34.8) | 178 (32.2) | |  | | 143 (27.0) | 141 (22.9) | 284 (24.8) |  | 143 (24.3) | 141 (21.9) | 284 (23.0) |
| *Unemployed* | 33 (4.1) | 44 (5.0) | 77 (4.5) |  | | 18 (6.4) | | 23 (8.5) | 41 (7.4) | |  | | 15 (2.8) | 21 (3.4) | 36 (3.1) |  | 15 (2.6) | 21 (3.3) | 36 (2.9) |
| *Housewife/househusband* | 21 (2.6) | 40 (4.5) | 61 (3.6) |  | | 7 (2.5) | | 8 (3.0) | 15 (2.7) | |  | | 14 (2.6) | 32 (5.2) | 46 (4.0) |  | 14 (2.4) | 32 (5.0) | 46 (3.7) |
| *Retired* | 514 (63.4) | 543 (61.4) | 1057 (62.3) |  | | 163 (58.0) | | 133 (49.3) | 296 (53.7) | |  | | 351 (66.2) | 410 (66.7) | 761 (66.5) |  | 351 (59.7) | 410 (63.6) | 761 (61.7) |
| *Retired early* | 30 (3.7) | 22 (2.5) | 52 (3.1) |  | | 7 (2.5) | | 10 (3.7) | 17 (3.1) | |  | | 23 (4.3) | 12 (2.0) | 35 (3.1) |  | 23 (3.9) | 12 (1.9) | 35 (2.8) |
| *Permanently incapacitated for work* | 18 (2.2) | 27 (3.1) | 45 (2.7) |  | | 10 (3.6) | | 13 (4.8) | 23 (4.2) | |  | | 8 (1.5) | 14 (2.3) | 22 (1.9) |  | 8 (1.4) | 14 (2.2) | 22 (1.8) |
| ***Diseases ^a^*** *(multiple choices possible)* |  |  |  |  | |  | |  |  | |  | |  |  |  |  |  |  |  |
| *Myocardial infarction* | 114 (14.1) | 103 (11.6) | 217 (12.8) |  | | 35 (12.5) | | 26 (9.6) | 61 (11.1) | |  | | 79 (14.9) | 77 (12.5) | 156 (13.6) |  | 85 (14.5) | 82 (12.7) | 167 (13.5) |
| *Stroke* | 75 (9.2) | 74 (8.4) | 149 (8.8) |  | | 24 (8.5) | | 22 (8.1) | 46 (8.3) | |  | | 51 (9.6) | 52 (8.5) | 103 (9.0) |  | 53 (9.0) | 53 (8.2) | 106 (8.6) |
| *Metabolism disorder* | 455 (56.1) | 510 (57.6) | 965 (56.9) |  | | 141 (50.2) | | 139 (51.5) | 280 (50.8) | |  | | 314 (59.2) | 371 (60.3) | 685 (59.8) |  | 344 (58.5) | 383 (59.4) | 727 (59.0) |
| *Angina pectoris* | 107 (13.2) | 117 (13.2) | 224 (13.2) |  | | 41 (14.6) | | 31 (11.5) | 72 (13.1) | |  | | 66 (12.5) | 86 (14.0) | 152 (13.3) |  | 71 (12.1) | 90 (14.0) | 161 (13.1) |
| *Lung disease* | 124 (15.3) | 147 (16.6) | 271 (16.0) |  | | 53 (18.9) | | 39 (14.4) | 92 (16.7) | |  | | 71 (13.4) | 108 (17.6) | 179 (15.6) |  | 79 (13.4) | 117 (18.1) | 196 (15.9) |
| *Heart Failure* | 128 (15.8) | 131 (14.8) | 259 (15.3) |  | | 48 (17.1) | | 35 (13.0) | 83 (15.1) | |  | | 80 (15.1) | 96 (15.6) | 176 (15.4) |  | 87 (14.8) | 101 (15.7) | 188 (15.2) |
| *Hypertension* | 569 (70.2) | 656 (74.1) | 1225 (72.2) |  | | 198 (70.5) | | 192 (71.1) | 390 (70.8) | |  | | 371 (70.0) | 464 (75.4) | 835 (72.9) |  | 409 (69.6) | 482 (74.7) | 891 (72.3) |
| *Diabetes* | 214 (26.4) | 223 (25.2) | 437 (25.8) |  | | 75 (26.7) | | 51 (18.9) | 126 (22.9) | |  | | 139 (26.2) | 172 (28.0) | 311 (27.2) |  | 151 (25.7) | 182 (28.2) | 333 (27.0) |
| *Cancer* | 78 (9.6) | 77 (8.7) | 155 (9.1) |  | | 27 (9.6) | | 15 (5.6) | 42 (7.6) | |  | | 51 (9.6) | 62 (10.1) | 113 (9.9) |  | 55 (9.4) | 64 (9.9) | 119 (9.7) |
| ***Drugs ^a^*** *(multiple choices possible)* |  |  |  |  | |  | |  |  | |  | |  |  |  |  |  |  |  |
| *Antihypertensive agents* | 596 (73.5) | 657 (74.2) | 1253 (73.9) |  | | 199 (70.8) | | 189 (70.0) | 388 (70.4) | |  | | 397 (74.9) | 468 (76.1) | 865 (75.5) |  | 440 (74.8) | 489 (75.8) | 929 (75.3) |
| *Platelet function inhibitor* | 654 (80.6) | 716 (80.9) | 1370 (80.8) |  | | 210 (74.4) | | 199 (73.7) | 409 (74.2) | |  | | 444 (83.8) | 517 (84.1) | 961 (83.9) |  | 491 (83.5) | 540 (83.7) | 1031 (83.6) |
| *Statins* | 470 (58.0) | 513 (58.0) | 983 (58.0) |  | | 148 (52.7) | | 133 (49.3) | 281 (51.0) | |  | | 322 (60.8) | 380 (61.8) | 702 (61.3) |  | 356 (60.5) | 397 (61.6) | 753 (61.1) |
| ***Revascularization ^a^*** |  |  |  |  | |  | |  |  | |  | |  |  |  |  |  |  |  |
| *Yes* | 257 (31.7) | 242 (27.3) | 499 (29.4) |  | | 75 (26.7) | | 60 (22.2) | 135 (24.5) | |  | | 182 (34.3) | 182 (29.6) | 364 (31.8) |  | 192 (32.7) | 185 (28.7) | 377 (30.6) |
| *No* | 422 (52.0) | 512 (57.9) | 934 (55.1) |  | | 154 (54.8) | | 172 (63.7) | 326 (59.2) | |  | | 268 (50.6) | 340 (55.3) | 608 (53.1) |  | 310 (52.7) | 364 (56.4) | 674 (54.7)) |
| *No information provided* | 132 (16.3) | 131 (14.8) | 263 (15.5) |  | | 52 (18.5) | | 38 (14.1) | 90 (16.3) | |  | | 80 (15.1) | 93 (15.1) | 173 (15.1) |  | 86 (14.6)) | 96 (14.9) | 182 (14.8) |
| ***Group heart rate training ^a^*** |  |  |  |  | |  | |  |  | |  | |  |  |  |  |  |  |  |
| *Yes* | 110 (13.6) | 111 (12.5) | 221 (13.0) |  | | 31 (11.0) | | 23 (8.5) | 54 (9.8) | |  | | 79 (14.9) | 88 (14.3) | 167 (14.6) |  | 85 (14.5) | 90 (14.0) | 175 (14.2) |
| *No* | 684 (84.3) | 754 (85.2) | 1438 (84.8) |  | | 244 (86.8) | | 242 (89.6) | 486 (88.2) | |  | | 440 (83.0) | 512 (83.3) | 952 (83.1) |  | 492 (83.7) | 538 (83.4) | 1030 (83.5) |
| *No Information provided* | 17 (2.1) | 20 (2.3) | 37 (2.2) |  | | 6 (2.1) | | 5 (1.9) | 11 (2.0) | |  | | 11 (2.1) | 15 (2.4) | 26 (2.3) |  | 11 (1.9) | 17 (2.6) | 28 (2.3) |
| ***Nationality ^a^*** |  |  |  |  | |  | |  |  | |  | |  |  |  |  |  |  |  |
| *German* | 763 (94.1) | 833 (94.1) | 1596 (94.1) |  | | 265 (94.3) | | 248 (91.9) | 513 (93.1) | |  | | 498 (94.0) | 585 (95.1) | 1083 (94.6) |  | 498 (84.7) | 585 (90.7) | 1083 (87.8) |
| *Other* | 20 (2.5) | 21 (2.4) | 41 (2.4) |  | | 9 (3.2) | | 10 (3.7) | 19 (3.4) | |  | | 11 (2.1) | 11 (1.8) | 22 (1.9) |  | 11 (1.9) | 11 (1.7) | 22 (1.8) |
| *No information provided* | 28 (3.5) | 31 (3.5) | 59 (3.5) |  | | 7 (2.5) | | 12 (4.4) | 19 (3.4) | |  | | 21 (4.0) | 19 (3.1) | 40 (3.5) |  | 79 (13.4) | 49 (7.6) | 128 (10.4) |

^a^ Categorical variables: n (%)

^b^ Quantitative variables: M (SD)

*Information on education, income, marital status, number of children, nationality and occupation was collected at baseline only, and was not available from the 88 participants who completed only the 12-month follow-up questionnaire. These patients were included in no information provided.
