# Supplementary material for: Measuring walking impairment in patients with intermittent claudication: psychometric properties of the Walking Estimated-Limitation Calculated by History (WELCH) questionnaire
Source: PeerJ. 2021 Aug 26;9:e12039. doi: 10.7717/peerj.12039 (PMC8415277; doi:10.7717/peerj.12039)
Supplement: Supplemental Information 2 [file peerj-09-12039-s002.docx]

|  | **(Visual) inspection** | *WELCH/WIQ walking distance* | *WELCH/WIQ walking speed* | *WELCH/WIQ stair climbing* | *WELCH/WIQ total* | *WELCH/VASCUQOL activity* | *WELCH/ GAD7* | **Diagnostics/ Solution when violated** |
| --- | --- | --- | --- | --- | --- | --- | --- | --- |
| ***“Each score on X (Y) should be independent of other X (Y) scores”*** |  | Related to study design | | | | | |  |
| ***“Scores on both X and Y should be quantitative and normally distributed”*** | Q-Q Plot, histogram | WELCH: deviation from normality  Walking distance: deviation from normality | WELCH: deviation from normality  Walking speed: approximately normal | WELCH: deviation from normality  Stair climbing: approximately normal | WELCH: deviation from normality  WIQ total: normal | WELCH: deviation from normality  Activity: approximately normal | WELCH: deviation from normality  GAD7: extremely skewed | Pearson’s r is robust to deviations from normality (Havlicek et al., 1976); can be neglected if N > 30 (Agresti et al., 2009); no correction |
| ***“Scores on Y should be linearly related to scores on X”*** | Scatterplot | Approximately linear | Approximately linear | Approximately linear | Approximately linear | Approximately linear | Approximately linear | - |
| ***“X, Y scores should have a bivariate normal distribution”*** |  | Assumed if both variables each show univariate normality | | | | | |  |
| ***No extreme outliers*** |  | WELCH: 14 outliers  WIQ: no outliers | WELCH: 14 outliers  WIQ: no outliers | WELCH: 14 outliers  WIQ: no outliers | WELCH: 14 outliers  WIQ: no outliers | WELCH: 14 outliers  VASCUQOL: 3 outliers | WELCH: 14 outliers  GAD7: 14 outliers | Sensitivity analyses without outliers shows only marginal correlation difference; no deletion of outliers |

Testing of Assumptions

**Correlation**

**Independent-Samples T-Test**

|  | **(Visual) inspection** | WELCH/ high vs low QoL (both t0) | **Diagnostics/ Solution when violated** |
| --- | --- | --- | --- |
| ***Quantitative, approximately normally distributed (within each group)*** | Q-Q Plot, histogram | Deviation from normality in low QoL group, high group approximately normal | Welch’s t-test is robust to deviations from normality (Delacre et al., 2017); can be neglected if N > 30 (Agresti et al., 2009); no correction |
| ***Homogeneity of variances*** | Levene’s test | Violated | Welch’s t-test with *degrees of freedom adjusted for unequal* variances |
| ***Independent observations both between and within groups*** |  | Related to study design | - |
| ***No extreme outliers*** | Boxplots | Violated | Sensitivity analyses without outliers shows only marginal difference; no deletion of outliers |

**Multiple Linear Regression**

|  | **(Visual) inspection** | WELCH score predicted from BMI, age, income, comorbid diseases, medication, gender, education, revascularization and heart rate training | **Diagnostics/ Solution when violated** |
| --- | --- | --- | --- |
| ***Normal distribution of residuals*** | Histogram | Approximately normal | - |
| ***Linear Relationship between the outcome variable and the independent variables*** | Scatterplot | Age/WELCH: approximately linear  BMI/WELCH: approximately linear  Dummy variables met assumption of linearity by definition | - |
| ***Absence of multicollinearity*** | Variance Inflation factor (VIF) | For all values VIF < 2 indicating the absence of multicollinearity | - |
| ***Homogeneity of variances*** | Scatterplot (standardized residuals vs. Predicted values) | Violated | Logarithmic transformation (base 10) of the dependent variable |
| ***No extreme outliers*** | Boxplots | Violated | Sensitivity analyses without outliers shows only marginal difference in regression results; no deletion of outliers |

**Intraclass Correlation Coefficient**

|  | **(Visual) inspection** | WELCH score at 12-month follow-up/ WELCH retest score | **Diagnostics/ Solution when violated** |
| --- | --- | --- | --- |
| ***Normal distribution*** | Q-Q Plot, Histogram | Approximately normal | - |
| ***Homogeneity of variances*** | Scatterplot | No heterogeneity of variances | - |

**ROC Curve**

|  | **(Visual) inspection** |  | **Diagnostics/ Solution when violated** |
| --- | --- | --- | --- |
| ***The cut-off score chosen to discriminate between the population showing improvements and the population not showing any improvements is the same in both groups.*** | - | Assumption is met | - |

**References**

- Agresti, A., & Finlay, B. (2009). *Statistical methods for the social sciences*. Upper Saddle River, N.J: Pearson Prentice Hall.
- Delacre, M., Lakens, D., & Leys, C. (2017). Why psychologists should by default use Welch's t-test instead of student's t-test. International Review of Social Psychology, 30(1), 92-101.
- Havlicek, L. L., & Peterson, N. L. (1976). Robustness of the Pearson correlation against violations of assumptions. *Perceptual and Motor Skills, 43*(3), 1319–1334.
