# Supplementary material for: Measuring walking impairment in patients with intermittent claudication: psychometric properties of the Walking Estimated-Limitation Calculated by History (WELCH) questionnaire
Source: PeerJ. 2021 Aug 26;9:e12039. doi: 10.7717/peerj.12039 (PMC8415277; doi:10.7717/peerj.12039)
Supplement: Supplemental Information 4 [file peerj-09-12039-s004.pdf]

Für jede der folgenden drei Fragen sollten Sie nur eine Antwort (von den 8 möglichen Antwortmöglichkeiten) auswählen.

Wie lange können Sie ohne Unterbrechung auf ebener Strecke laufen (oder glauben Sie, dass Sie könnten):

	gar nicht	30 Sekunden	1 Minute	3 Minuten	10 Minuten	30 Minuten	1 Stunde	3 Stunden & mehr
Wenn Sie langsam gehen? (Langsamer als Ihre Familie, Freunde oder Gleichaltrige)	❑	❑	❑	❑	❑	❑	❑	❑
Wenn Sie mit mittlerer Geschwindigkeit gehen? (Mit derselben Geschwindigkeit wie Ihre Familie, Freunde oder Gleichaltrige)	❑	❑	❑	❑	❑	❑	❑	❑
Wenn Sie schnell gehen? (Schneller als Ihre Familie, Freunde oder Gleichaltrige)	❑	❑	❑	❑	❑	❑	❑	❑

Zur Beantwortung der folgenden Frage sollten Sie nur eine Antwortmöglichkeit auswählen.

Im Vergleich zur durchschnittlichen Gehgeschwindigkeit von Ihren Verwandten, Freunden und Menschen in Ihrem Alter, gehen Sie in der Regel ...

❑ … viel langsamer

❑ … langsamer

❑ … ein wenig langsamer

❑ … mit der gleichen Geschwindigkeit

❑ … schneller
